# Molecular Cloning and Characterization of Estrogen-Related Receptor Gene in *Corbicula fluminea*: Expression Profiles in Response to Bisphenol A and Its Substitutes Exposure

**DOI:** 10.3390/biology14101384

**Published:** 2025-10-10

**Authors:** Ruiyi Xu, Weili Guo, Pengyu Zhang, Chunnuan Zhang

**Affiliations:** The Water Environment and Animal Safety Laboratory, Henan University of Science and Technology, Luoyang 471023, China; 15517948598@163.com (W.G.); 13333881963@163.com (P.Z.); zhangchunnuan@haust.edu.cn (C.Z.)

**Keywords:** bisphenol analogs, bivalves, estrogen-related receptor, RACE cloning

## Abstract

**Simple Summary:**

Bisphenol A (BPA) and its alternatives have been identified as endocrine-disrupting chemicals (EDCs), yet there is a paucity of research data regarding their reproductive disrupting effects on mollusks. In this study, we successfully cloned the full length of the estrogen-related receptor (ERR) gene from *Corbicula fluminea* (*Cf*ERR) by RACE technology. Subsequently, we conducted bioinformatics analysis and tissue-specific expression analysis of *Cf*ERR. Additionally, we examined the expression profiles of the *Cf*ERR gene following 28-day exposure to 1, 10, and 100 μg/L concentrations of 17β-estradiol (E2), BPA, and its substitutes (bisphenol S (BPS), bisphenol F (BPF), and bisphenol AF (BPAF)). Sequence alignment revealed that the *Cf*ERR showed high homology with those of other bivalve mollusks. Tissue expression analysis demonstrated that the *Cf*ERR gene exhibited the highest expression level in the gonad. Results from the exposure experiment indicated that the expression of *Cf*ERR was upregulated under the stress of all tested pollutants. This study provided a scientific basis for the conservation of shellfish germplasm resources, laying a foundation for investigating the reproductive regulation mechanism of bivalve mollusks and offering important data support for the toxic assessment of BPA and its alternatives.

**Abstract:**

Bisphenol A (BPA) and its substitutes have been identified as endocrine-disrupting chemicals (EDCs). However, little information is available on their reproductive endocrine disruptive effects in mollusks. This study cloned the full-length sequence (2434 bp) of the estrogen-related receptor (ERR) gene in the freshwater bivalve *Corbicula fluminea* and performed a bioinformatics analysis and tissue-specific expression analysis. We further examined the expression of the *Cf*ERR gene after exposure to E2, BPA, and their substitutes (BPS, BPF, and BPAF) at 1, 10, and 100 μg/L for 0, 1, 7, 14, 21, and 28 days. The results showed that *Cf*ERR is a nuclear protein with a typical structure. Phylogenetic analysis indicated a high degree of similarity among bivalve species. The high expression of *Cf*ERR in the gonad suggested its important role in reproductive regulation. The exposure experiment confirmed that *Cf*ERR showed a time- and dose-dependent upregulation in response to all pollutants, with BPS and BPAF exhibiting stronger estrogenic interference effects. This study facilitates a better understanding of the reproductive regulation of bivalves and provides data to support the toxicity evaluation of BPA and its substitutes.

## 1. Introduction

Endocrine-disrupting chemicals (EDCs) are a class of exogenous substances that could affect the synthesis and metabolism of endogenous hormones in humans and aquatic animals [1]. Bisphenol A (BPA), as a typical bisphenol-type endocrine disruptor, was widely used in food packaging, bottles, dental sealants, and numerous aspects of daily consumer products and has a history of 50 years [2]. BPA, with its estrogenic interference effect, can cause damage to the reproductive endocrine system [3,4]. Currently, BPA has been controlled or banned worldwide, and substitutes with similar structures to BPA, such as bisphenol S (BPS), bisphenol F (BPF), and bisphenol AF (BPAF), have begun to flood the market [3]. Research has confirmed that BPS, BPF, and BPAF also have endocrine disruptive effects [4] and exhibit extremely high stability and persistence, with much higher bioavailability than BPA [5,6]. As a result, these substitutes are often detected in aqueous media and show an increasing occurrence trend [7,8]. In recent years, the reproductive endocrine disruptive effects of BPA and its substitutes on aquatic organisms have attracted widespread attention.

In vertebrates, estrogen receptors (ERs) are considered the main target of EDCs, and EDCs could competitively bind to ER by mimicking 17β-estradiol (E2) and activating ER pathways to produce endocrine-disrupting effects [9]. The E2 also plays an important reproductive activity in invertebrates; studies on male and female soft-shell clam *Mya arenaria* and female octopuses *Octopus vulgaris* have shown that the concentration of E2 fluctuates with the whole reproductive cycle [10,11]. The rate of vitellogenin synthesis and gamete maturation was positively correlated with the injected E2 of oysters *Magallana gigas* [12]. After injecting E2 into the scallop *Mizuhopecten yessonsis*, it can increase its production of gamete cells [13]. In vitro experiments have found that ER of octopuses *O. vulgaris* has binding activity with E2 and estrone, and 10 pM E2 can induce significant upregulation of ER gene expression [14]. However, the tertiary protein structure of ERs in mollusks differs greatly from that of vertebrates; there is no consensus regarding the mechanism by which E2 regulates reproductive function through activating ERs in mollusks.

The estrogen-related receptor (ERR) belongs to the group of nuclear receptors (NRs), with high homology and similarity in structure and sequence to the DNA-binding domain of ERs, which also could bind to anthropogenic estrogenic ligands [15]. In humans, BPA strongly binds to ERRγ with high constitutive activity while showing low affinity to ER [16]. In aquatic vertebrates, high expression of the ERR gene has been determined in the gonads of Japanese medaka *Oryzias latipes* and killifish *Fundulus heteroclitus* [17,18]. Tohyama et al. (2015) found that in vitro recombinant expression of ERα had a binding activity with BPA that was 0.017–0.1 times that of E2 in five fish species [19]. And 200 μg/mL BPS could induce significant upregulation of ER and Vtg genes in female zebrafish [20]. BPS and BPF, as well as BPA, can disrupt ERR, Vtg, and VtgR transcription in the brackish water flea *Diaphanosoma celebensis* [21]. In mollusks, studies have shown that ER and ERR genes play important roles in gonadal maturation of freshwater snails *Marisa cornarietis* [22]. A study of mussels *Mytilus edulis* and *Mytilus galloprovincialis* indicated that both ER and ERR expressions in gonadal cells responded to estrogenic compounds [23]. In addition, tetrabromobisphenol A (TBBPA) and 4-nitrophenol (4-NP) have been shown to activate the ER-recombinant yeast system in oysters *M. gigas* and clams *Ruditapes philippinarum* [24]. A recent study found that the ER gene expressions in three bivalve mollusks responded to BPA and its substitutes exposure [25]. The ERRs provide a new perspective for the supplement of ERs on reproduction, while there is still a lack of knowledge regarding the reproductive regulation of ERRs in mollusks and their response to EDC exposure.

*Corbicula fluminea*, one of the dominant species of benthic bivalves in freshwater ecosystems, has abundant natural resources and is suitable for studying the reproductive endocrine disruption effects of EDCs [26]. In this study, the ERR gene was first cloned from *C. fluminea* (*Cf*ERR), and bioinformatics analysis was conducted, and then the tissue-specific expression of the ERR gene was measured. In order to contribute to a better understanding of the endocrine-disruption mechanism, a 28-day exposure experiment to BPA and its substitutes (BPS, BPF, and BPAF) of *C. fluminea* was also investigated. All of these studies will help to gain insights into the reproductive interference mechanisms of BPA and its substitutes in mollusks.

## 2. Materials and Methods

### 2.1. Experimental Animals and Pollutants Preparation

The test animal (*C. fluminea*) was a one-year-old mature individual, hermaphroditic, which was reared in the freshwater culture system of the Aquatic Environment Toxicology Laboratory of the College of Animal Science and Technology of Henan University of Science and Technology. No residual bisphenol pollutants were found in their bodies. Small amounts of chlorella powder were added in the morning and evening for feeding (3 g/m^3^). The *C. fluminea* (shell length 2.21 ± 0.2 cm) were reared in 22 cm × 16 cm × 17 cm tanks; the volume of the aquaculture water body is 4 L. The experimental water was tap water that had been aerated for 24 h, and it is completely replaced every day. The breeding conditions were as follows: water temperature of 20 ± 2 °C, salinity of 1%, and pH value of 7.0. Record the water quality parameters each time you change the water. For gene cloning and tissue-specific expression analysis, 6 and 9 *C. fluminea* individuals were sampled per replicate, respectively. After 7 days of temporary rearing, 6 *C. fluminea* were collected from the control group, and their gonadal tissues were taken for gene cloning. Additionally, 9 *C. fluminea* were collected, and their gonads, mantle film, gills, digestive cecum, and adductor muscles were sampled using liquid nitrogen grinding. Each tissue sample was accurately weighed to 0.1 g for tissue-specific expression analysis.

Dissolve BPA (purity ≥ 99%), BPS (≥99%), BPF (≥98.0%), BPAF (≥99.0%), and natural estrogen E2 (≥97.0%), respectively, in ≤0.005% (*v*/*v*) dimethylsulphoxide (DMSO, purity 99.5%) [27]. Then, dilute it with deionized water to prepare the mother solution, and the concentration of the mother solution is 0.04 g/L. Every 7 days, a new batch of pollutant mother solution is prepared and stored in brown glass bottles. All the standard substances and organic reagents used in the experiment were purchased from Thermo Fisher in Shanghai, China. Set up blank control and DMSO control groups, and for each pollutant, set the exposure group concentrations as 1, 10, and 100 μg/L, respectively. Each group has 3 replicates, totaling 66 tanks. During routine water renewal, the mother solution of the contaminant was added to maintain a constant contaminant concentration in the water. The exposure lasts for 28 days. Samples are taken at 0, 1, 7, 14, 21, and 28 days. For each concentration, 9 individuals of *C. fluminea* are taken, the gonads are removed, immediately frozen in liquid nitrogen, ground in a grinding instrument, and used for fluorescence quantitative experiments.

### 2.2. RACE Cloning of CfERR Gene

#### 2.2.1. RNA Extraction and First-Strand cDNA Synthesis

Total RNA was extracted using Trizol reagent (Invitrogen Company, Waltham, MA, USA). After concentration treatment, using oligo (dT) as the primer, add SMARTer II Oligonucleotide and SMARTScribe reverse transcriptase to synthesize the first strand of cDNA. The SMARTer II Oligonucleotide provides specific sequences to introduce the universal primer binding sites required for subsequent PCR.

#### 2.2.2. The 5′ and 3′ RACE Experiment

The primers were designed using Primer Premier 5.0 software (Premier, Ottawa, ON, Canada), and the sequences were sent to Shanghai Sangon Biotech Co., Ltd (Shanghai, China). for the synthesis of the primers. Specific primers GSP1–GSP3 were designed (Table 1). The first-strand cDNA of the target gene was synthesized using SUPERSCRIPT II RT enzyme and primer GSP-1 on the total RNA. The synthesized cDNA was then subjected to RNA removal treatment using RNase Mix. The synthesized cDNA was then purified using GLASSMAX, and a dC tail was added to it using terminal deoxynucleotidyl transferase (TdT) enzyme. First-round PCR was performed with GSP2 and anchor primer AAP under the following conditions: 94 °C for 2 min; 35 cycles of 94 °C for 30 s, 55 °C for 30 s, and 72 °C for 1 min; and 72 °C for 10 min. Nested PCR was performed using first-round products. The amplification products were extracted and purified using the Nucelo Spin Gel and PCR Clean-Up Kit (Takara Bio, San Jose, CA, USA). Target bands were purified, ligated into the pMD18-T vector, transformed into competent Escherichia coli cells, and positive clones were sequenced.

The 3′ RACE primers GSP1-G2 were designed. Reverse transcription was performed using SMARTScribe™ Reverse Transcriptase (Takara Bio, San Jose, CA, USA) and 3′ CDS Primer A. First-round PCR was conducted using GSP1 and the universal primer mix (UPM). The PCR reaction procedure is as follows: pre-denaturation at 94 °C for 2 min, 94 °C for 30 s, 72 °C for 3 min, repeat this cycle 5 times; 94 °C for 30 s, 70 °C for 30 s, 72 °C for 3 min, 5 cycles; 94 °C for 30 s, 68 °C for 30 s, 72 °C for 1 min, 27 cycles. Second-round PCR was then performed using GSP2 and UPM. The reaction conditions and procedures are the same as those in the first round. The target bands from the second-round PCR were recovered and sequenced.

#### 2.2.3. Sequence Assembly and Open Reading Frame (ORF) Prediction of the *Cf*ERR

The full-length cDNA sequence of *Cf*ERR was assembled from 5′ and 3′ RACE results. ORF prediction was performed, which yielded the complete cDNA sequence of *Cf*ERR.

### 2.3. Bioinformatics of CfERR

ORF Finder was used to predict the start and stop codons; the amino acid sequence was translated using the Novopro website (https://www.novopro.cn/tools/ (accessed on 25 July 2023)). Protein BLAST (https://blast.ncbi.nlm.nih.gov/Blast.cgi?PROGRAM=blastp&PAGE_TYPE=BlastSearch&LINK_LOC=blasthome (accessed on 10 May 2024)) was used to analyze the homology of *Cf*ERR with ERRs from other species. The ProtParam website (https://web.expasy.org/protparam/ (accessed on 15 May 2024)) was used to characterize the physical and chemical properties of the *Cf*ERR protein. The Simple Modular Architecture Research Tool (https://smart.embl.de/ (accessed on 20 May 2023)) was used to predict the conserved domains of *Cf*ERR. MEGA 11 (Institute for Genomics and Evolutionary Medicine (iGEM), Philadelphia, PA, USA) software was used to perform multiple sequence alignment (using the ClustalW algorithm) and phylogenetic analysis (using the neighbor-joining (NJ) method with 1000 bootstrap replicates). An evolutionary tree was constructed based on the Poisson correction model using the amino acid sequences of ERRs from multiple species, including mollusks, crustaceans, and vertebrates.

### 2.4. Tissue-Specific Analysis of the CfERR

The qRT-PCR was performed using cDNA from *C. fluminea* tissues, including gonads, mantle film, gills, digestive cecum, and adductor muscles. Specific primers were designed based on the ERR sequence; *β-actin* was used as a reference. The SYBR PrimeScript™ RT kit (Hunan accurate biotechnology engineering Co., Ltd., Changsha, China) was used for amplification (primers in Table 1). Amplification conditions were as follows: initial denaturation at 94 °C for 30 s; 40 cycles of denaturation at 95 °C for 5 s and annealing/extension at 60 °C for 30 s; and a final hold at 12 °C.

### 2.5. Expression of the CfERR Gene Under Long-Term Exposure to BPA and Its Substitutes

Samples were taken at 0, 1, 7, 14, 21, and 28 days, respectively. Total RNA was extracted from the *C. fluminea* gonad tissues of the blank control group, the DMSO control group, and the experimental groups under different pollutant stress at different concentrations. Reverse transcription reagents were used to synthesize cDNA. Specific primers for *Cf*ERR were designed using Primer Premier 5 software, with *β-actin* of *C. fluminea* as the internal reference gene. The expression of ERR mRNA was detected by SYBR-qPCR (primers are shown in Table 1).

### 2.6. Statistical Analyses

Expression was analyzed via the 2^−ΔΔCT^ method [28]. All data are presented based on the relative mRNA expression levels, namely the mean and its standard deviation (SD). A one-way ANOVA analysis of variance was used to conduct a difference analysis between different concentration experimental groups and the control group at the same time. The results were subjected to consistency analysis, and they were consistent with the conclusions of the variance analysis and followed Tukey’s test to determine the differences between different treatments. All data were analyzed using SPSS 29 software (SPSS Inc., Chicago, IL, USA). * (*p* < 0.05) indicates a significant difference, ** (*p* < 0.001), and *** (*p* ≤ 0.001), which means a statistically extremely significant difference.

## 3. Results

### 3.1. Cloning and Bioinformatics Analysis of the CfERR

#### 3.1.1. Sequence Analysis of the *Cf*ERR

**Figure 1:**
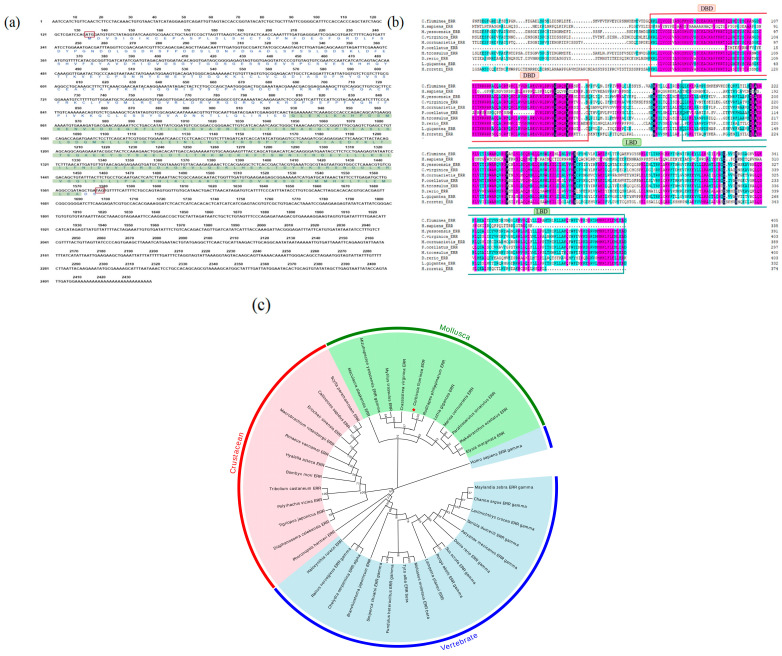
Bioinformatics analysis of the *Cf*ERR *gene*. (**a**) The cDNA sequence of *Cf*ERR cloned by RACE and the predicted amino acid sequence encoded by it. (**b**) Multiple sequence alignment of *Cf*ERR with ERRs from other species. (**c**) Phylogenetic tree constructed based on the amino acid sequences of ERRs from different species. The target gene *Cf*ERR is marked with a red star in the figure to indicate its location. Numbers at branching points represent bootstrap values. The mollusks, crustaceans, and vertebrates are highlighted with red, green, and blue backgrounds, respectively. GenBank sequences in the tree include: Mollusca: *Elysia marginaata* ERR: GFR92274.1, *Plakobranchus ocellatus* ERR:GF048275.1, *Parafossarulus striatulus* ERR:AVN57016.1, *Marisa cornuarietis* ERR:ABI97118.1, *Lottia gigantea* ERR:AGG68283.1, *Ruditapes philippinarum* ERR:AYJ00904.1, *Crassostrea virginica* ERR:XP_022328830.1, *Mytilus trossulus* ERR:NC_086375.1, *Mizuhopecten yessoensis* ERR gamma:ABI97120.1, *Maculaura alaskensis* ERR:APU51308.1. Crustacean: *Scylla paramamosain* ERR:ADB43256.1, *Callinectes sapidus* ERR:UKB93139.1, *Eriocheir sinensis* ERR: AXU37748.1, *Macrobrachium rosenbergii* ERR:AOY10609.1, *Penaeus vannamei* ERR:ROT74072.1, *Hyalella azteca* ERR:KAA0195992.1, *Bombyx mori* ERR:ANS60466.1, *Tribolium castaneum* ERR:APU51308.1, *Polyrhachis vicina* ERR:ABR88112.1, *Tigriopus japonicus* ERR:AID52852.1, *Diaphanosoma celebensis* ERR:QJE49261.1, *Phoronopsis harmeri* ERR:QVG60147.1. Vertebrate: *Halocynthia roretzi* ERR:ABO42263.1, *Rattus norvegicus* ERR gamma:NP_976081.1, *Chelydra serpentina* ERR alpha:KAG6939449.1, *Branchiostoma japonicum* ERR:BAR91683.1, *Siniperca chuatsi* ERR gamma:WGW15304.1, *Fundulus heteroclitus* ERR gamma:XP_0012736989.1, *Tyto alba* ERR beta:APQ40581.1, *Molossus molossus* ERR beta:KAF6499948.1, *Lampetra planeri* ERR:QIM58148.1, *Pongo abelii* ERR gamma:NP_001125696.1, *Sus scrofa* ERR gamma:ALS35337.1, *Danio rerio* ERR gamma:XP_005158828.1, *Astyanax mexicanus* ERR gamma:XP_007240543.2, *Seriola dumerili* ERR gamma:XP_022599138.1, *Larimichthys crocea* ERR gamma:KKF26670.1, *Channa argus* ERR gamma:KAF3702758.1, *Maylandia zebra* ERR gamma:XP_004539576.1, *Homo sapiens* ERR gamma:AAQ93380.1.

Based on the transcriptomic data of *C. fluminea*, a core fragment of the *Cf*ERR gene (849 bp) was identified. Using RACE technology, the full-length cDNA sequence of the ERR gene from *C. fluminea* was successfully cloned, measuring 2434 bp. The ORF of the *Cf*ERR sequence is 1443 bp in length and encodes 480 amino acids (Figure 1a). The cloned *Cf*ERR sequence has been submitted to the NCBI database, and the assigned GenBank accession number is OR248152. Analysis using ProtParam website showed that the theoretical molecular weight of this polypeptide is 54.2 kDa, the theoretical isoelectric point (pI) is 5.90, the grand average of hydropathicity (GRAVY) is −0.410, and the aliphatic index is 80.61. The instability index is 49.15.

Online analysis with PSORT II prediction (http://www.genscript.com/tools/psort (accessed on 18 May 2024)) indicated that the *Cf*ERR protein is a nuclear protein (52.2%). The nuclear localization signal (NLS) recognition regions are located within the amino acid sequences KRRR and RRRK. The DNA-binding region signature of nuclear hormone receptors is CLVCGDIASGFHYGVSSCEACKAFFKR. No N-terminal signal peptide is found in the *Cf*ERR protein. In this study, the complete amino acid sequence of the *Cf*ERR protein and its ligand-binding domain (LBD) is compared with the corresponding sequences of ERR proteins from other species. The nucleotide sequence and predicted amino acid sequence of *Cf*ERR are shown in Figure 1a.

#### 3.1.2. Homology and Phylogenetic Analysis of the *Cf*ERR

To investigate the similarity between *C. fluminea* and other species, the amino acid sequence of *Cf*ERR and its LBD were compared with the corresponding sequences of ERR proteins from mollusca: *R. philippinarum*, *C. virginica*, *M. trossulus*, *M. yessoensis*, *P. ocellatus*, *L. gigantea*, *M. cornuarietis*, and vertebrates: *H. sapiens*, *D. rerio*. As shown in Figure 1b, the amino acid sequences and LBDs of these ERR proteins exhibit high similarity. Among the analyzed species, *R. philippinarum* showed the highest similarity to *Cf*ERR. Compared with the full-length amino acid sequence of *Cf*ERR, its LBD sequence demonstrates higher homology with the LBDs of ERR proteins from other mollusks.

Phylogenetic analysis revealed three main clades of ERRs in the phylogenetic tree: one consisting of crustaceans, one of mollusks, and one of vertebrates. Figure 1c illustrates the major branches of ERRs among crustaceans, mollusks, and vertebrates, suggesting that these three groups of ERRs may represent evolutionary differentiation. Multiple sequence alignments show that the sequence identity of ERR among different species varies depending on structural differences. Furthermore, the amino acid sequence homology of ERRs among different species also varies due to structural variations.

**Figure 2:**
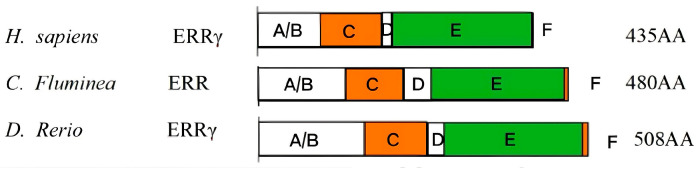
Comparison of the ERR domains. The boxed region indicates regions that are corresponding to the translated protein regions. Letters A–F indicate the locations of the ERR domains.

**Figure 3:**
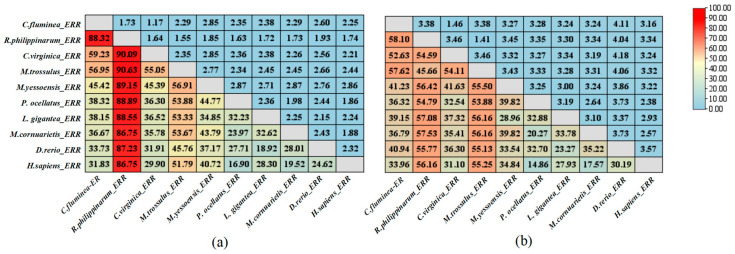
Amino acid sequence similarity of *Cf*ERR to ERR proteins from other species. (**a**) Genetic distances between the complete amino acid sequence of *Cf*ERR and those of ERR proteins from other species. (**b**) Genetic distances between the LBD sequence of *Cf*ERR and the LBD sequences of ERR proteins from other species, including mollusca (*R. philippinarum* ERR, *C. virginica* ERR, *M. trossulus* ERR, *M. yessoensis* ERR, *P. ocellatus* ERR, *L. gigantea* ERR, *M. cornuarietis* ERR), and vertebrates (*D. rerio* ERR and *H. sapiens* ERR).

Based on the 480 amino acids encoded by *Cf*ERR, it was confirmed that *Cf*ERR has the typical domain organization of the NR family, including an N-terminal A/B domain, a DNA-binding C domain (DBD), a hinge D domain, a ligand-binding E domain, and a C-terminal F domain (Figure 2). According to the genetic distance calculation results shown in Figure 3, the full-length amino acid sequence and LBD sequence of *Cf*ERR exhibit higher similarity (>55%) to those of *M. yessoensis*, *P. ocellatus*, and *M. trossulus*, while showing lower similarity to those of the vertebrates *H. sapiens* and *D. rerio*.

### 3.2. Tissue-Specific Expression Analysis of CfERR

**Figure 4:**
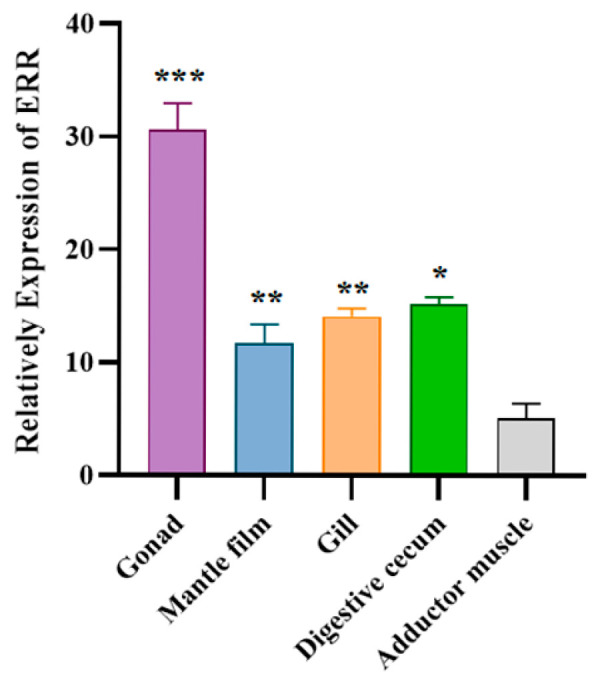
Expression of ERR in different tissues of *C. fluminea*. The data are presented as mean ± SD (the number of replicate experiments for each treatment was three). The asterisks indicate significant differences in ERR gene expression (using one-way ANOVA followed by Tukey’s test, * indicates *p* ≤ 0.05, ** indicates *p* ≤ 0.01, *** indicates *p* ≤ 0.001).

The tissue-specific expression analysis results of ERR are shown in Figure 4, indicating that the ERR gene was detectable in all tested tissues of *C. fluminea*. The highest relative expression was observed in the gonad, while the lowest was found in the adductor muscle. The relative expression of ERR in the gonad was approximately six times higher than that in the adductor muscle.

### 3.3. Expression of the CfERR Gene Under 28-Day Exposure to BPA and Its Substitutes

**Figure 5:**
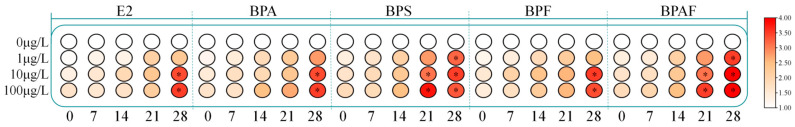
The *Cf*ERR gene expression under E2 and BPA and its substitutes exposure. The results are presented as mean ± SD. At the same time compared with the control group, there was a statistically significant difference, marked by an asterisk (* *p* < 0.05).

The exposure experiment revealed no significant differences between the DMSO control group and the blank control group. The relative expression levels of *Cf*ERR mRNA in the gonads are shown in Figure 5. The experiment utilized one-way analysis of variance to compare the expression levels of *Cf*ERR at different exposure concentrations at the same time point with those of the control group to determine whether there was a significant difference. Under pollutant stress, the expression level of *Cf*ERR mRNA gradually increased with exposure time. At the same point, the relative expression level of *Cf*ERR mRNA significantly increased with increasing pollutant concentration (*p* < 0.05), showing a significant dose-dependent upregulation. Moreover, BPA and its substitutes exerted an upregulatory effect on the expression of *Cf*ERR on *Cf*ERR. Among them, BPS and BPAF showed stronger estrogenic effects than BPA and BPF.

## 4. Discussion

### 4.1. The Evolutionary Analysis of ERRs in Bivalve Mollusks

This study successfully cloned the full-length cDNA sequence of *Cf*ERR and confirmed that the protein encoded by *Cf*ERR is a nuclear protein (with a nuclear localization probability of 52.2%). At present, two subfamilies of ERs, ERα and Erβ, have been found [29], and three subtypes of ERRs (ERRα, ERRβ, and ERRγ) have been identified in mammals [30]. However, only one type of ER and ERR have been found in mollusks. Phylogenetic analysis indicated that *Cf*ERR clusters within the invertebrate clade and showed high homology with ERRs from other mollusks. It shows the highest similarity with the clam *R. philippinarum* ERR, which is also a bivalve species; this result further verifies the accuracy of the *Cf*ERR sequence cloning (Figure 1). Currently, full-length cDNA of ERs has been cloned in six shellfish, including one scallop species (*Chlamys farreri*), two oyster species (*Crassostrea angulata* and *M. gigas*), and two mussel species (*M. edulis*, *M. galloprovincialis*) and *C. fluminea*. In addition, full-length cDNA of ERR-homologous genes has been cloned in the scallop *Mizuhopecten yessoensis*, the oyster *Crassostrea virginica*, and two mussels (*M. edulis*, *M. galloprovincialis*) and the clam *R. philippinarum* [23,25,31,32,33].

In humans, ERRs, along with ERs, belong to subfamily 3 (NR3) of the nuclear receptor (NR) superfamily, and ERRs and ERs share structural features of NRs, and their gene and amino acid sequences exhibit high homology [34]. Through amino acid domain analysis, the *Cf*ERR also has DBDs with P-box, D-box, and NLS structures (Figure 2), which is consistent with the typical characteristics of the ER family. Based on the genetic distance results (Figure 3), the LBD sequence of *Cf*ERR has a lower similarity of less than 40% with both humans and zebrafish and a higher similarity with invertebrates, indicating the different reproductive regulatory mechanisms of mollusks and vertebrates. The mollusks ERR may have lower sensitivity to vertebrate E2 and may possess new structural endogenous E2-like molecules.

### 4.2. The Tissue-Specific Expression Analysis of ERRs in Bivalve Mollusks

In this experiment, *Cf*ERR expression was detected in all tested tissues of *C. fluminea*, indicating ERR might have various functions in different tissues, and the expression of *Cf*ERR was in the order of gonads > digestive cecum > gills > mantle film > adductor muscle (Figure 4), which was consistent with a study in clams *R. philippinarum* [35]. In mollusks, ERRs were highly expressed in gonads of mussels (*M. edulis*), two squid species (*Sepiella japonica* and *Sepia latimanus*) [36,37]. However, a study of the female snail *Marisa cornuarietis* showed that the expression of ERR in the gonadal tissues was very weak [22]. The differences in ERR expression among gonads of different mollusk species may be attributed to differences in their reproductive stages or inherent reproductive traits between *C. fluminea* and *M. cornuarietis*, while the low-level expression of ERR may also be the key to its reproductive function [23]. Research on the function of ERRs in chordates suggested that their roles include controlling cell proliferation and differentiation, lipid storage and consumption, and mitochondrial biosynthesis in the brain. The function of ERRs in chordates may have been acquired through evolutionary preservation, suggesting that *Cf*ERR might also be involved in life processes [38]. Research has confirmed that ERRγ regulates testicular steroidogenesis in mouse Leydig cells [20]. Evolutionary tree analysis showed that the *Cf*ERR has high homology with ERRγ of other species, suggesting that *Cf*ERR might be involved in the regulation of reproductive endocrine. However, the mechanism of ERR involvement in reproductive endocrine regulation in invertebrates needs further exploration.

### 4.3. Effects of EDCs on ERR Expression in Bivalve Mollusks

EDCs disrupt the normal hormone-mediated pathways by mimicking endogenous hormones and binding to hormonal receptors competitively, leading to adverse effects on offspring reproduction and development, reproductive neurotoxicity, mutagenesis, and cancer [39]. BPA could interact with hormonal receptors such as ER and result in endocrine-disrupting effects, leading to adverse outcomes to the reproductive system [40]. Additionally, research has found that BPA binds more strongly to human ERRγ than to ERs [41]. And bivalve ERRs are localized in the gonads of both male and female Mytilus, and they function by binding to the ERR response element (ERRE) to regulate gametogenesis [23,42]. Recently, BPA and its substitutes have received worldwide concern because of the toxic effects on aquatic species, and the substitutes have been determined to be more toxic than BPA in some species [43]. Two benzene rings in the BPS molecule are connected by a sulfonyl group (-SO_2_-) and are relatively more stable [44]. Moreover, studies have reported that BPAF can affect the expression of the key reproductive genes ERα, 3βhsd, and vtg2 in marine medaka (*Oryzias melastigma*), causing disruption of ovarian development and a decrease in sperm quantity [45].

This study found that the expressions of *Cf*ERR were upregulated under exposure to BPA and its substitutes (Figure 5); similarly, the ERR gene expression was upregulated significantly following short-term exposure to BPA and 4-nitrophenol (NP) in the midge *Chironomus riparius* [46]. And BPA induced a significant increase in ER and ERR mRNA expression of the freshwater snail *Physa acuta*, indicating the pollutant is involved in similar reproductive regulation events as the receptors in mollusks [47]. In addition, BPA and its substitutes of BPS and BPF modulated mRNA expressions of ERR, Vtg, and VtgR, which caused the adverse effect to the reproduction pathway in brackish water flea *Diaphanosoma celebensis* [21]. The above research confirmed that BPA and its substitutes had estrogenic effects, and ERR genes in mollusks are also important targets of EDCs as well. However, further clarification is needed on the endocrine disruption mechanism of EDCs on mollusks.

## 5. Conclusions

This study successfully cloned the ERR gene from *C. fluminea* using RACE technology, and the cloned sequence was submitted to the NCBI database. Through sequence alignment and genetic distance analysis, it was found that the LBD sequence of the *C. fluminea* has higher similarity with that of mollusks and is highly homologous to the ERR of bivalves. The analysis of tissue-specific expression showed that ERR had the highest expression level in the gonadal tissue of *C. fluminea*. A 28-day exposure experiment to BPA and its substitutes showed that the expression level of the *Cf*ERR gene was upregulated at all tested concentrations (1, 10, and 100 μg/L). BPS and BPAF produced a stronger upregulatory effect on *Cf*ERR expression than BPA and BPF. This study demonstrates that the ERR gene plays an important regulatory role in the reproductive development of *C. fluminea*, thus laying a foundation for further investigating the reproductive toxicity of bisphenol-based EDCs in bivalves.

## Figures and Tables

**Table 1:** Primers used in RACE cloning and qPCR experiments.

Purpose	Primer Name	Sequence (5′ to 3′)
5′ RACE	B947-1(GSP1)	GTTTTATTGTCTGCGA
	B947-2(GSP2)	TTTGTACGAATGGAAATGAA
3′ RACE	B947-3(GSP3)	GCACCCTGTCCAACCTCA
	C583-1 (GSP1)	GAAACTCCACGAGGCTCTCACCGA
	C583-2(GSP2)	TAATGGTGGCCTCAAACGTGTCGG
qRT-PCR	*Cf*ERR-S	AAAAACTCAAAGCCCACC
	*Cf*ERR-A	GCTCAGACTCGCAAACC
qRT-PCR	*β-actin*-S	GGCTGTGCTTTCATTGT
	*β-actin*-A	TTTCTCTTTCGGCTGTT

## Data Availability

The relevant data of this experiment will be provided proactively if necessary.
